# Ruptured Basilar Artery Blood Blister-Like Aneurysm Treated Using a Single Low-Profile Visualized Intraluminal Support (LVIS) Stent: A Case Report

**DOI:** 10.7759/cureus.101196

**Published:** 2026-01-09

**Authors:** Takuya Maeda, Haruhiko Kikuta, Takao Kojima, Yui Mano, Masazumi Fujii

**Affiliations:** 1 Department of Neurosurgery, Fukushima Medical University, Fukushima, JPN; 2 Department of Neurosurgery, Chubu Rosai Hospital, Nagoya, JPN

**Keywords:** basilar artery, blood blister-like aneurysm, dissecting aneurysm, lvis stent, subarachnoid hemorrhage

## Abstract

A basilar artery blood blister-like aneurysm (BBA) is a rare entity for which a standard treatment has not yet been established. We report a case of basilar artery BBA in the subacute stage of subarachnoid hemorrhage (SAH) that was treated using a single low-profile visualized intraluminal support (LVIS) stent. A 63-year-old woman presented with sudden-onset posterior neck pain. Head computed tomography (CT) revealed modified Fisher group 3 SAH, extending from the prepontine and basal cisterns to the quadrigeminal cistern. The patient was diagnosed with Hunt and Hess grade 2 SAH. Initial digital subtraction angiography (DSA) revealed an aneurysm in the right internal carotid artery (ICA), which was treated with coil embolization. Although a minute bulge on the basilar artery was identified on the initial three-dimensional DSA (3D DSA), it was too subtle for a definitive diagnosis on day 1. Repeat DSA was performed on day 11 because hemorrhage from the posterior circulation could not be excluded. DSA on day 11 revealed a 1.7-mm projection on the dorsal wall of the basilar artery by 3D DSA, confirming a basilar artery BBA. Dual antiplatelet therapy (DAPT) was initiated, and endovascular treatment was performed. The BBA was extremely small with a pinhole neck, making stent-assisted coil embolization difficult and high-risk for rupture; therefore, a single LVIS stent was placed in the basilar artery, forming a dense stent mesh around the aneurysm. The postoperative course was uneventful, and the patient was discharged without neurological deficit. Four months later, follow-up DSA showed complete resolution of the BBA. If coil placement is challenging in basilar artery BBA, treatment with a single LVIS stent may be an effective alternative option.

## Introduction

A basilar artery blood blister-like aneurysm (BBA) is a rare entity that is generally considered a subtype of dissecting aneurysm (DA) [1]. In a large Japanese case series, basilar artery DAs (BADAs) accounted for only 0.37% of all ruptured intracranial aneurysms or DAs [2]. It has been estimated that BADAs comprise approximately 12% of basilar artery aneurysms [3], whereas the proportion of basilar artery BBAs is thought to be even lower. In both BBA and BADA, surgical or endovascular internal trapping is challenging because preservation of the basilar artery is required. However, advances in neck-bridging and flow diverter (FD) stents have enabled reconstructive endovascular treatments that preserve the parent artery. Moreover, favorable outcomes have been reported with single or overlapping stenting, stent-assisted coil embolization, or flow diversion for intracranial BBA and BADA [2-12].

We report a case in which the source of bleeding could not be identified on initial digital subtraction angiography (DSA). A tiny aneurysm was subsequently detected on repeat DSA, confirming a basilar artery BBA, which was successfully treated using a single low-profile visualized intraluminal support (LVIS) stent.

## Case presentation

**Figure 1:**
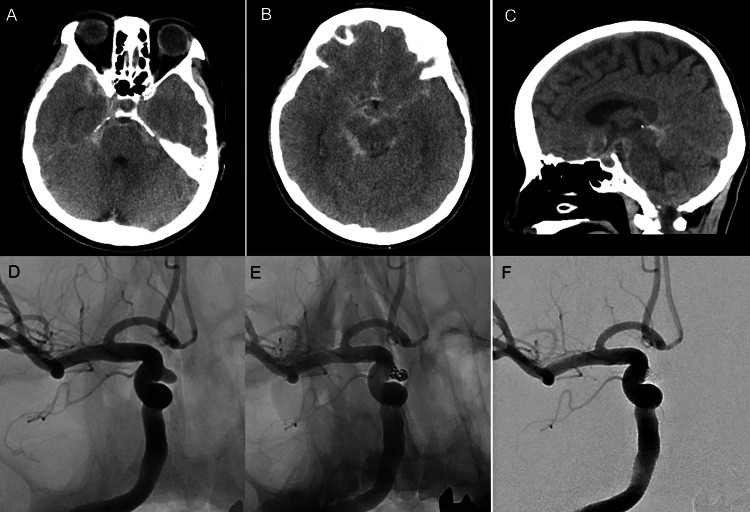
Initial head computed tomography (CT) and intra-aneurysmal coil embolization. (A-C) Head CT on admission. Subarachnoid hemorrhage extended from the prepontine and basal cisterns to the quadrigeminal cistern. (D) Right internal carotid artery (ICA) angiography on admission revealed a right internal carotid artery-superior hypophyseal artery (ICA-SHA) aneurysm. The ICA-SHA aneurysm was considered to have ruptured. (E, F) Subsequently, balloon-assisted coil embolization was performed, resulting in complete obliteration.

**Figure 2:**
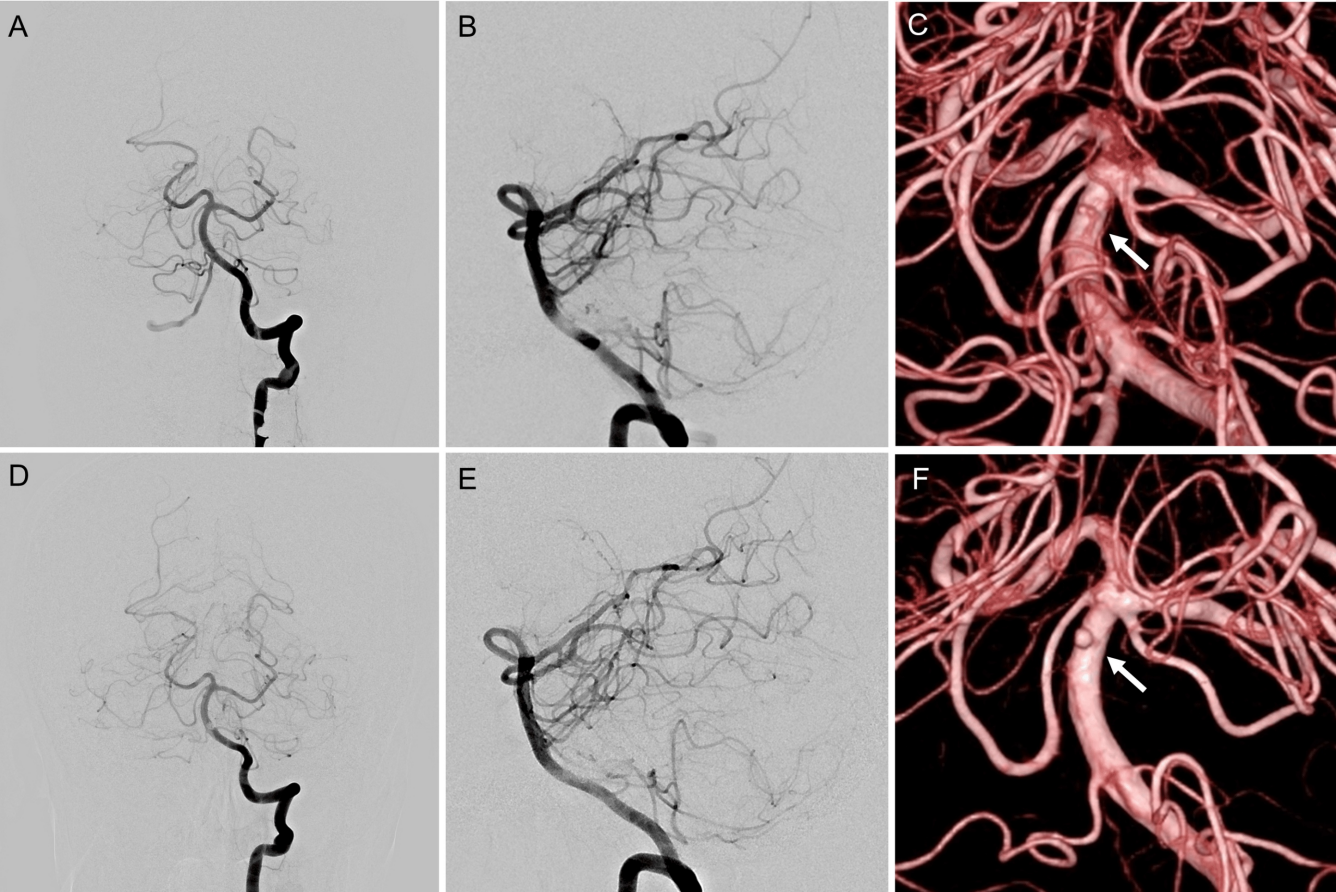
Left vertebral artery angiography (VAG) on admission and on day 11. (A-C) Left VAG on admission. (A, B) No aneurysm was noted in the anteroposterior or lateral view. (C) Three-dimensional digital subtraction angiography (3D DSA) demonstrated a minute bulge on the dorsal wall of the basilar artery (arrow). (D-F) Left VAG on Day 11. (D, E) No changes in conventional anteroposterior and lateral views. (F) 3D DSA revealed a non-branching basilar trunk aneurysm, with a maximum diameter of 1.6 mm and height of 1.7 mm (arrow). The diagnosis was a blood blister-like aneurysm on the dorsal basilar trunk.

A 63-year-old woman with a history of breast cancer was brought to another hospital because of sudden-onset posterior neck pain. She was diagnosed with subarachnoid hemorrhage (SAH) of unknown etiology based on head computed tomography (CT) and three-dimensional CT angiography (3D CTA) findings and was referred to our hospital the following day (day 1). On presentation, her only symptom was headache, with no neurological deficits. SAH was graded as Hunt and Hess grade 2 and World Federation of Neurosurgical Societies (WFNS) grade I. Head CT demonstrated Fisher group 3 and modified Fisher group 3 SAH occupying the prepontine and basal-to-quadrigeminal cisterns (Figure 1A-1C). DSA performed on day 1 revealed a medially protruding right internal carotid artery (ICA)-superior hypophyseal artery (SHA) aneurysm (Figure 1D). The aneurysm measured 3.0 × 2.7 mm in diameter, with a height of 3.1 mm and a neck width of 2.9 mm. Although the distribution of SAH suggested bleeding from the posterior circulation, only a minute bulge was observed on the dorsal wall of the basilar artery on 3D DSA, and this finding was not considered significant at that time (Figure 2A-2C). Therefore, only the right ICA-SHA aneurysm was treated on day 1. An 8-Fr Optimo EPD balloon guide catheter (Tokai Medical Products, Aichi, Japan) was positioned distal to the cervical segment of the right ICA, and a 6-Fr Navien distal access catheter (Medtronic, MN, USA) was advanced coaxially into the horizontal segment of the cavernous ICA (Fisher C4 segment). Through the 6-Fr Navien catheter, a Scepter C 4 × 10-mm balloon catheter (Terumo Neuro, CA, USA) was positioned near the aneurysmal neck, and an Excelsior SL-10 microcatheter (Stryker, MI, USA) was navigated into the aneurysm using a Chikai 0.014-inch microguidewire (Asahi Intecc, Aichi, Japan). Coil embolization was performed using the balloon neck remodeling technique with deployment of three coils (Figure 1E, 1F).

**Figure 3:**
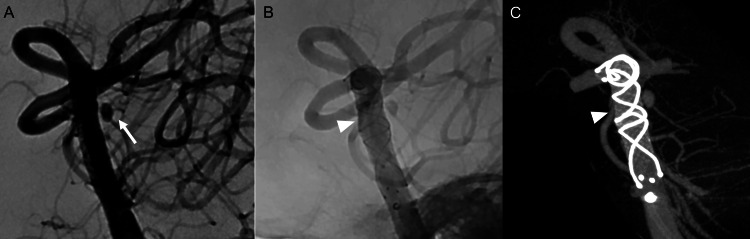
Second intraoperative view. Intraoperative view on day 22. (A) Preoperative subtraction angiography showed a basilar trunk blood blister-like aneurysm (arrow) projecting in the right posterior-lateral direction. The aneurysm neck was the size of a pinhole. (B) Postoperative angiography showing a single low-profile visualized intraluminal support (LVIS) stent deployed from the left posterior cerebral artery to the lower segment of the basilar artery. From the distal to proximal direction, the LVIS stent was deployed, and the mesh was densely placed in the aneurysm bulge (arrowhead). (C) 3D maximum intensity projection image just after the LVIS stent was deployed. The dense stent mesh was observed near the aneurysmal neck to increase metal coverage (arrowhead).

Postoperatively, the patient did not develop cerebral vasospasm or hydrocephalus. On day 11, follow-up DSA confirmed complete obliteration of the ICA-SHA aneurysm; however, a small aneurysm measuring 1.6 mm in diameter, 1.7 mm in height, with a pinhole neck, was identified on the dorsal wall of the basilar artery (Figure 2D-2F). A diagnosis of SAH due to rupture of a BBA was established, and endovascular treatment in the subacute stage was selected. Dual antiplatelet therapy (DAPT; aspirin 100 mg/day and clopidogrel 75 mg/day) was initiated on Day 12. The P2Y12 reaction unit (PRU), measured using the VerifyNow system (Accumetrics, CA, USA), was 199 on day 22, and endovascular treatment was performed on the same day. Given the extremely small size and low height of the aneurysm, as well as its very narrow neck, microcatheter navigation and coil placement were considered technically difficult and associated with a high risk of perforation. In addition, to avoid occlusion of perforating branches arising from the basilar artery trunk, treatment with a single LVIS (Terumo Neuro) stent without coil embolization was selected. A 7-Fr Fubuki guiding catheter (Asahi Intecc, Aichi, Japan) was positioned distal to the V2 segment of the left vertebral artery. Through this catheter, a Headway 21 microcatheter (Terumo Neuro, CA, USA) was advanced into the left posterior cerebral artery (P1 segment) using a Chikai 0.014-inch microguidewire (Asahi Intecc, Aichi, Japan). A 4 × 22-mm LVIS stent was deployed from the P1 segment to the mid-basilar artery, just distal to the origin of the anterior inferior cerebellar artery, creating a dense stent mesh at the aneurysm neck (Figure 3). Only a single LVIS stent was deployed. DSA performed 10 minutes after stent placement demonstrated slight contrast retention within the aneurysm. To minimize the risk of perforator occlusion, the procedure was concluded after placement of the single LVIS stent, with the expectation of gradual aneurysm thrombosis.

The postoperative course was uneventful. The patient underwent rehabilitation and was discharged on day 45 with a modified Rankin Scale (mRS) score of 0, without ischemic or hemorrhagic complications.

**Figure 4:**
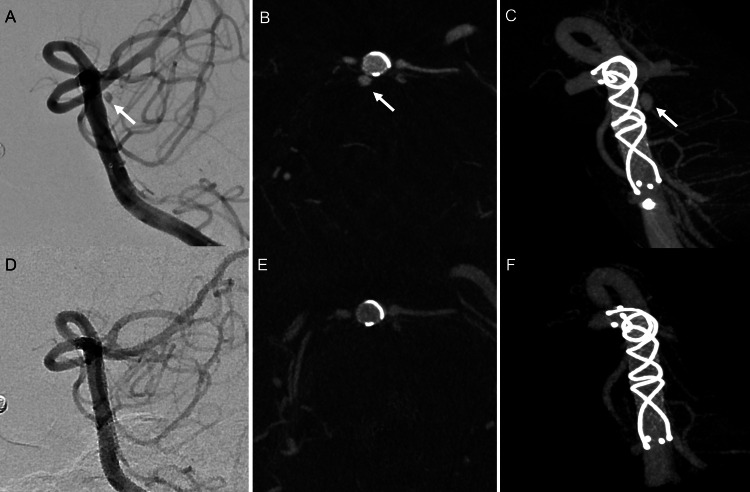
Left vertebral artery angiography just after stent placement and four months after stenting. (A-C) Immediately after low-profile visualized intraluminal support (LVIS) stent placement for the basilar artery blood-blister aneurysm (BBA). (A) The BBA was visualized (arrow). Left vertebral artery digital subtraction angiography at the working angle. (B) Cone-beam CT axial image and (C) 3D maximum intensity projection image. (D-F) Four months after stenting, the BBA had been completely obliterated. Panels D-F correspond to A-C, respectively.

DSA performed four months after onset confirmed complete obliteration of the basilar artery BBA (Figure 4). Clopidogrel was subsequently discontinued, and antiplatelet therapy was continued with aspirin alone. Follow-up has been continued at our outpatient clinic.

## Discussion

Differentiating between basilar artery BBA and DA

BBA is broadly considered a subtype of DA. Mizutani et al. classified non-atherosclerotic spindle-shaped or dissecting cerebral aneurysms into four types [1]: Type 1, a classic DA characterized by widespread disruption of the internal elastic lamina (IEL), which angiographically presents as a fusiform aneurysm with an irregular wall and stenotic portion near the proximal or distal end; Type 2, a segmental ectasia characterized by stretched and fragmented IEL with intimal thickening, which angiographically presents as a fusiform aneurysm with a smooth contour; Type 3, a dolichoectatic DA characterized by fragmented IEL and multiple dissections of thickened intima with luminal thrombus, which angiographically presents as a tortuous fusiform appearance with irregular contrast caused by the luminal thrombus; and Type 4, a saccular aneurysm arising from the arterial trunk, exhibiting minimal disruption of the IEL without intimal thickening, which angiographically appears unrelated to the branching zones.

BBA is considered Mizutani Type 4, whereas DA, with the pearl and string sign, irregular dilatation, and stenosis or occlusion of the arterial trunk, is considered Mizutani Type 1. In the present case, no irregular fusiform dilatation or stenosis of the basilar trunk itself was observed, and a prominent saccular aneurysm was present at a non-branching site of the trunk, consistent with Mizutani Type 4, i.e., BBA.

In general, BBA refers to a saccular aneurysm at a non-branching site on the anterior wall of the ICA [6]. However, basilar artery BBA is uncommon, and some previous reports have grouped it with basilar artery DAs or basilar trunk aneurysms [3,5,9]. Caution is therefore required during diagnosis and literature review. Moreover, during treatment, in cases of DA (Mizutani Type 1), coiling of the dilated lesion (dissecting site) is often performed with stenting. However, in cases of BBA (Mizutani Type 4), it may be a pseudoaneurysm formed at a pinhole in the arterial trunk, and inserting a microcatheter or coiling may carry a high risk of rerupture. Distinguishing the pathology is important for appropriate treatment.

Diagnosis of basilar artery BBA

BBA is not always obvious in the early stages of rupture, and a reported one-third of cases go undetected on initial DSA [6]. Furthermore, when an aneurysm is present at another site, it may be treated while the BBA goes undetected, resulting in fatal rerupture [13]. If the SAH distribution at onset indicates a dense posterior fossa or intraventricular hemorrhage, bleeding from the posterior circulation should be taken into consideration, and repeat DSA should be performed [14]. In the present case, SAH extended from the prepontine and basal cisterns to the quadrigeminal cistern on initial head CT, and bleeding from the posterior circulation could not be excluded. A minute bulge on the dorsal wall of the basilar artery was identified only using 3D DSA on initial DSA (day 1) (Figure 2A-2C); however, it could not be determined as the source of hemorrhage. On day 11, a repeat 3D DSA revealed that the bulge had enlarged into an overt aneurysm (likely a pseudoaneurysm), resulting in a definitive diagnosis of BBA.

As ruptured aneurysmal walls are enhanced on black-blood gadolinium-enhanced T1-weighted imaging (T1WI), this technique is useful for identifying the bleeding source in non-traumatic non-aneurysmal SAH [15]. In the present case, early use of this method may have confirmed that the right ICA aneurysm had not ruptured, and that the bleeding originated from the basilar artery BBA.

Treatment strategy and stent selection

Although it is uncertain in basilar artery BBA, rebleeding is a poor prognostic factor in BADA, and acute treatment may be desirable in basilar artery BBA, if possible [2,3]. Following the development from the neck-bridging stent to the FD stent, reconstructive endovascular therapy for both basilar artery BBA and DA has been reported to prevent rebleeding and provide good outcomes [2-12]. Therefore, even in the acute stage of SAH, stent-assisted coiling (SAC) or stenting with DAPT may be viable treatment options in the current clinical consensus. On the other hand, SAC and FD in the acute stage of SAH accounted for 19.4% and 17.8% of ischemic and hemorrhagic complications, respectively [16,17]. Therefore, it is also reasonable to initiate antiplatelet therapy and endovascular treatment after exiting the acute stage, as in the present case. In this patient, the timing of treatment was determined by carefully balancing the risks associated with acute stage DAPT and potential perforator infarction against the patient’s neurological stability and the absence of early rebleeding.

Regarding the choice of stent, the overlapping stent with two LVIS stents has a higher flow diversion effect than the Pipeline FD stent (Medtronic) [18], and the effectiveness of this method has been previously demonstrated [11]. However, the ischemic complication rates associated with LVIS and FD in the posterior circulation have been reported as 4.8% and 11%, respectively [19,20]. The FD offers a higher flow diversion effect compared to single LVIS [18], but it was not selected because it carries a higher risk of perforating branches occlusion than single LVIS. On the other hand, laser-cut stents have the lowest flow diversion effect and a lower risk of perforating branch occlusion than single LVIS [18]; however, they were not selected in the present case because they are least likely to block blood flow into the aneurysm without coiling. In the present case, the intra-aneurysmal coiling would have been very difficult, with a high risk of perforation because of the small size and pinhole neck. Therefore, to achieve aneurysm occlusion and avoid compromise of perforating branches, only a single LVIS was selected.

Regarding technique, when deploying the LVIS, pushing and creating a dense metal mesh could potentially stress the dissecting wall. However, since the pseudoaneurysm had a pinhole neck, microcatheter placement was considered riskier, so we opted to pack the stent mesh around the aneurysm only, in order to close the aneurysmal neck to prevent occlusion of perforating branches (Figure 3B, 3C). In further detail, the LVIS stent was initially deployed from the posterior cerebral artery to the basilar artery apex using a standard microcatheter unsheathing technique. Once the stent reached the basilar artery trunk where the BBA was located, the stent mesh was specifically compacted at the aneurysm neck by pushing the delivery shaft during deployment. Proximal to the neck, the remainder of the stent was fully deployed using the simple unsheathing technique to avoid compromising the perforating arteries originating from the basilar artery.

The coil placement for BBA, pseudoaneurysm, sometimes carries a high risk. Yamamura et al. also reported coil deviation into the extra-aneurysmal space immediately after SAC [12]. For BBA, coil embolization may have a high risk of perforation, especially if the aneurysm is tiny with a low aspect ratio, and stenting alone may also be an alternative treatment option, as it was in our case.

Although the successful treatment of BBA or DA with an FD stent has been reported [3-5,9], the type of stent and the combination of stent and coil are selected on a case-by-case basis, considering the geometry of the aneurysm, the preferences of the institution, and the surgeon [2,3]. At present, the optimal method among SAC, overlapping stent, and FD stent remains unclear; thus, future accumulation of cases and prospective studies is required.

Comparison with previous reports

To the best of our knowledge, there have been 12 reported cases of basilar artery BBA with a confirmed treatment course, including ours [4,5,7-12] (Table 1). Nine patients were female, with a median age of 52 years (range: 42-68 years). Among the six cases for which the timing of the diagnosis was described, basilar artery BBA was identified on the initial DSA in only two cases (33%) [10,12]. In the remaining four cases, including ours, repeat DSA was required to identify the source of bleeding [5,11]. There was only one case of preoperative rebleeding [5]. All patients underwent DAPT before surgery, and neck-bridging stent alone, SAC, and FD were used in five, five, and four procedures, respectively. Two patients required additional treatment due to incomplete aneurysm occlusion after the first treatment [9,11]. On DSA performed three months or more after treatment, 11 patients had complete occlusion and 1 had a neck remnant. Only one case had an mRS score of 3 [9]; all others had scores of 0-1 from discharge to 27 months after treatment. Based on these previous cases, the rebleeding rate before radical treatment may have been lower than that in previously reported cases of basilar artery BBA (Table 1), and there was only one case of postoperative infarction [11]. Furthermore, the occlusion rates and functional outcomes were generally favorable. However, cases with poor clinical outcomes are likely underreported, and it cannot be concluded that the prognosis is generally favorable. Therefore, further accumulation of cases is desired.

**Table 1:** Cases of endovascular treatment for ruptured basilar artery blood blister-like aneurysms. H&H, Hunt and Hess, WFNS: World Federation of Neurosurgical Societies, BBA: blood blister-like aneurysm, mRS: modified Rankin Scale; N.A.: not available, SAC: stent-assisted coil, FD: flow diversion, NR: neck remnant, CO: complete obliteration. *Same patient as above; retreatment performed due to residual aneurysm. #Stent: neck bridging stenting. #FD: flow diverter stent.

Author (year)	Age	Sex	H&H grade	WFNS grade	Fisher group	Preoperative rebleeding	Date of BBA identified (days)	Treatment duration (days)	Treatment	Stent	Number of stents	Complication	Postoperative rebleeding	mRS	Aneurysmal occlusion 3M~	BBA size (mm)
Meckel et al. (2011) [10]	49	F	4	N.A.	3	None	0	6	SAC	N.A.	N.A.	None	None	1	NR	2.1 x 4.8
	44	F	3	N.A.	2	None	N.A.	N.A.	SAC	N.A.	N.A.	None	None	0	CO	2.0 x 3.0
Martin et al. (2012) [9]	42	F	2	I	3	None	N.A.	N.A.	Stent#	Enterprise	1	None	None	-	-	-
	42*	F	-	-	-	None	-	42	FD#	Pipeline	1	None	None	0	CO	2.2 x 2.3
Lim et al. (2013) [8]	45	F	2	N.A.	N.A.	None	N.A.	N.A.	SAC	N.A.	3	None	None	0	CO	N.A.
Kim and Ko (2014) [7]	52	M	2	N.A.	3	None	N.A.	N.A.	Stent	Neuroform + Enterprise	2	None	None	0	CO	2.1 x 2.0
Aydin et al. (2015) [4]	47	F	1	N.A.	N.A.	None	N.A.	15	FD	SILK	1	None	None	1	CO	2.0 x 2.5
	68	N.A.	2	N.A.	N.A.	None	N.A.	8	FD	SILK	1	None	None	0	CO	2.0 x 4.0
Derelle et al. (2016) [5]	62	F	2	I	4	Present	9	9	FD	Pipeline	1	None	None	1	CO	N.A.
Morinaga et al. (2019) [11]	52	M	4	IV	4	None	10	10	Stent	LVIS	2	None	None	3	CO	2
	62	F	2	I	3	None	5	5	Stent	LVIS	2	None	None	-	-	-
	62*	F	-	-	-	None	-	14	SAC	LVIS	1	Infarction	None	1	CO	1.7
Yamamura et al. (2021) [12]	53	F	3	N.A.	3	None	0	0	SAC	LVIS	1	None	None	0	CO	2.5 x 4.0
Present case	63	F	3	II	3	None	11	22	Stent	LVIS	1	None	None	0	CO	2.7 x 3.0

We considered BBA to be a subtype of BADA, and compared 2 large case series and 12 cases (Table 1). In a large BADA case series by Oya et al. [2], 17 (0.37%) of 4,586 SAH cases had a ruptured BADA. Eight patients were treated aggressively, while nine were treated conservatively. Three of the conservatively treated patients experienced rebleeding, and five died. The Glasgow Outcome Scale (GOS) scores at discharge were 4-5 in seven patients, 3 in four, and 1 in six. Although this case series was likely limited to BADA, patients with a low WFNS grade on admission generally had a better prognosis. However, all patients with rebleeding had a GOS score of 1 at discharge [2]. Saliou et al. [3] reported basilar trunk aneurysms in 52 of 2,522 patients (2.1%): Mizutani Type 1 in 6 patients (five ruptured); Type 2 in 11 patients (all unruptured); Type 3 in 22 patients (all unruptured); and Type 4 (likely including BBA) in 13 patients (five ruptured). Eight cases of ruptured BADA were treated aggressively, with a GOS score of 4-5 or an mRS of 0-1 in six patients and a GOS score of 1 in two. The prognosis was also better for patients with a low WFNS grade on admission [3].

Of the 12 reported cases of basilar artery BBA (Table 1), 11 patients had an mRS score of 0-1 at discharge or in the chronic stage. These favorable outcomes may be attributed to advancements in endovascular reconstructive techniques and stent devices. Aggressive treatment should be considered in treatable cases.

## Conclusions

We report a case of SAH due to rupture of a basilar artery BBA that was successfully treated with placement of a single LVIS stent in the subacute stage. BBAs may not present as a distinct bulge immediately after rupture; therefore, repeat DSA is essential when the source of hemorrhage is not identified on initial evaluation. In this context, 3D DSA is particularly useful for detecting extremely small lesions. Although endovascular treatment with stenting may be considered in the acute stage once a basilar artery BBA is identified, treatment in the subacute stage may represent a reasonable alternative, given the relatively high rates of ischemic and hemorrhagic complications associated with acute-stage stenting. Several cases of basilar artery BBA have been reported in which the bleeding source was initially unclear. As rebleeding from a BBA is associated with poor prognosis, repeat DSA and early identification of the bleeding source followed by definitive treatment are desirable whenever feasible. Although the indication is limited, placement of a single LVIS stent may be an effective treatment option for Mizutani Type 4 basilar artery BBAs that present as extremely small pseudoaneurysms with a pinhole neck.
